# Improved precision and accuracy for microarrays using updated probe set definitions

**DOI:** 10.1186/1471-2105-8-48

**Published:** 2007-02-08

**Authors:** Rickard Sandberg, Ola Larsson

**Affiliations:** 1 Massachusetts Institute of Technology, Department of biology, 68-211, Cambridge, MA 02139, USA; 2 University of Minnesota, Department of Medicine, MMC 276, Minneapolis, MN 55455, USA

## Abstract

**Background:**

Microarrays enable high throughput detection of transcript expression levels. Different investigators have recently introduced updated probe set definitions to more accurately map probes to our current knowledge of genes and transcripts.

**Results:**

We demonstrate that updated probe set definitions provide both better precision and accuracy in probe set estimates compared to the original Affymetrix definitions. We show that the improved precision mainly depends on the increased number of probes that are integrated into each probe set, but we also demonstrate an improvement when the same number of probes is used.

**Conclusion:**

Updated probe set definitions does not only offer expression levels that are more accurately associated to genes and transcripts but also improvements in the estimated transcript expression levels. These results give support for the use of updated probe set definitions for analysis and meta-analysis of microarray data.

## Background

Microarrays have been used for the last decade to analyze the global gene expression programs of different biological processes and disease states. During that time, e.g. the methodologies for background adjustment [1], normalization [2] and probe set summaries [3] have been improved and it is likely that further efforts will enable better analysis of microarray data. The exponential use of microarrays in biology has resulted in large public gene expression repositories that include thousands of arrays for the most commonly used Affymetrix platforms. The integrated analysis of this wealth of gene expression data raises both new possibilities and challenges [4].

Most of the commonly used Affymetrix platforms were designed before the respective genomes were fully sequenced. Therefore, these platforms have many probes that were designed after consensus sequences of clusters of Expressed Sequence Tags. In the original Affymetrix probe set definitions many probe sets often map to the same gene (e.g. they may target different transcript isoforms) and some integrative microarray studies use ad-hoc heuristics such as the average or maximum to integrate these values into a single expression estimate [5,6].

To curate and solve the above mentioned problems, updated probe set definitions have been generated by re-annotating the existing probes on Affymetrix platforms to better reflect the transcript information and gene annotations available today [7,8]. These pioneering studies have shown that updated probe set definitions will affect approximately 20–30% of all probe sets, thus affecting a large portion of the gene estimates [8]. As a consequence, the genes identified as differentially expressed using the original and updated probe set definition only show 50% overlap [7,8]. Updated probe set definitions that map probes to transcript annotations, such as ensEMBL transcripts, Refseq and Entrez GeneID are now available and can easily be integrated into bioconductor packages such as affy and gcrma. Updated probe annotations have also been shown to improve the cross-platform reproducibility of microarray experiments [9,10].

The use of updated probe set definitions represents a significant improvement in mapping the platform probe signals to genes, transcripts and even exon expression levels and will presumably become the standard procedure. There is however no study that has evaluated the impact of updated probe set definitions on precision and accuracy in the estimated expression levels. In this study we provide such a comparison and we show that updated probe set definitions have significantly better precision (reproducibility) and accuracy than the original probe set definitions. These results give support for a widespread use of updated probe set definition in analyzing and re-analyzing microarray data.

## Results

### Re-analyses of raw data using updated probe set definitions

We investigated how updated probe set definitions affect the estimated expression levels by re-analyzing a gene expression data set using both the original (NetAffx) and six updated probe set definitions (custom CDF's). Previously, this data set was used to estimate the precision and accuracy in microarray experiments across laboratories and platforms using the original probe set definitions [11]. The data set was generated by creating two RNA samples, which differed in the expression of only a few genes. Both samples were hybridized to two arrays each by five different labs. Within each lab the two pairs of replicates was used to estimate the precision and accuracy (see below) by analyzing the log2 relative expression level measurements between the two samples. Because five labs performed the identical experiment, this data set provides a good opportunity to study effects of selected probe set definitions, since the estimated precision and accuracy obtained in each lab can be summarized to provide a robust assessment of the effects. We therefore used this data set to address the effects of using six different recently published updated probe set definitions in comparison with the default probe set definitions provided by Affymetrix (NetAffx). We re-analyzed the raw data for the Affymetrix HG-U133A arrays generated in the five different laboratories to estimate probe set expression levels using six different updated probe set definitions [7] and using the default probe set definitions. The six different updated probe set definitions (custom CDF's) re-mapped the probes on the array to i. ensEMBL exons, ii. ensEMBL genes, iii. ensEMBL transcripts, iv. Entrez GeneIDs, v. Refseq transcripts and vi. UniGene ids [7].

### Significant improvement in precision using updated probe set definitions

We first investigated the effect of using updated probe set definitions on precision, which measures the data reproducibility and variability. As described previously [11], we defined precision as the correlation between the relative log2 expression ratios of the two RNA samples using the two pairs of replicates (i.e. A1/B1 vs A2/B2) pairs. The precision is a clear indication of the experiment performance and a correlation of 1 indicates perfect precision while a correlation of 0 indicates no precision. For each lab we calculated the precision using the different probe set definitions respectively. The mean precision difference for each updated probe set definition as compared with the original probe set definitions are reported in Table 1. The significance of each difference in precision was assessed by a two-tailed paired *t*-test using the precision differences obtained from the five labs. The precision was significantly improved for all updated probe set definitions except for the ensEMBL exons (Table 1), for which it was significantly worse (commented on below). The improvement was most obvious when using RMA estimated expression levels (Table 1).

**Table 1:** Improved precision using update probe set definitions

	**ensEMBL exon**	**ensEMBL gene**	**ensEMBL transcript_**	**Entrez**	**RefSeq**	**UniGene**
**MAS5**	-0.014 p = 0.094	0.013 p = 0.057	0.013 p = 0.0092	0.014 p = 0.056	0.019 p = 0.018	0.023 p = 0.052
**RMA**	-0.035 p = 0.0041	0.053 p = 0.00025	0.030 p = 0.0071	0.059 p = 0.00011	0.045 p = 0.00045	0.047 p = 2.9E-06
**GCRMA**	-0.11 p = 0.000051	0.023 p = 0.045	-0.0063 p = 0.28	0.040 p = 0.019	0.031 p = 0.062	0.028 p = 0.0071

We compared the precision in microarray experiments when using the updated probe set definitions in comparison with the original probe set definition (netAffx). We measured the precision as the Pearson correlation between two log2 ratios of expression levels comparing duplicate sample-vs-control measurements. The measurement indicates how well a fold change can be reproduced. Data from five different data sets generated using identical samples were averaged (mean) The table shows differences in means for updated probe set definitions compared to the original (NetAffx) definitions across the five different labs together with the p-value assessing the difference in means between each updated probe set definition compared to the original probe set definition (NetAffx) calculated using a paired t-test (two-tailed distribution). Three methods to generate expression estimates (Mas5, RMA and GCRMA) were compared.

**Figure 1:**
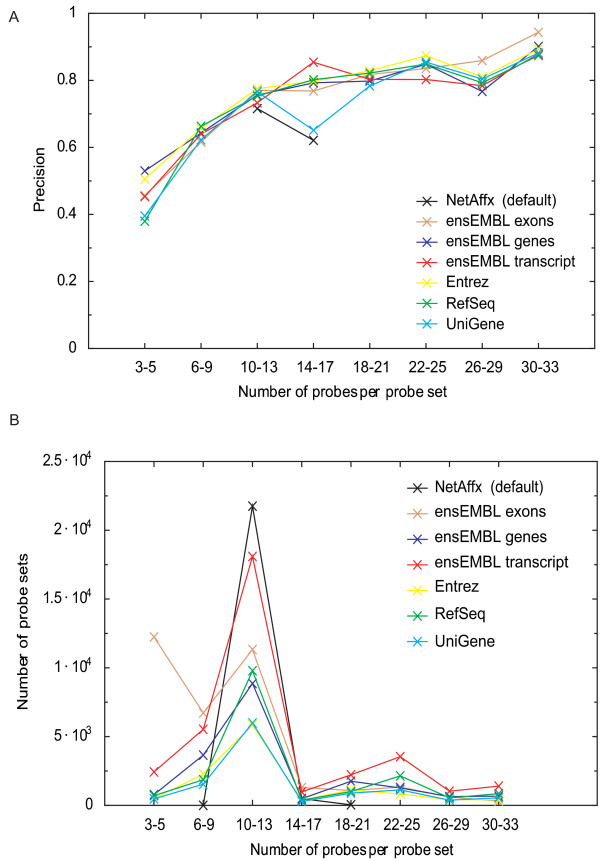
**Improved precision using updated probe set definitions**. (A) We compared the precision in microarray experiments when using the updated probe set definitions in comparison with the original probe set definition (netAffx). We measured the precision as the Pearson correlation between two log2 ratios of expression levels generated by comparing duplicate sample-vs-control comparisons. The measurement indicates how well a fold change can be reproduced. Data from five different data sets generated using identical samples (data normalized using RMA) were averaged (mean) [see Additional file 1] for similar figures for each laboratory independently). The x-axis shows the number of probes integrated into each probe set. We grouped the number of probes into intervals of three in order to obtain a sufficiently large number of probe sets to get a robust estimate of each data point. The y-axis shows the mean correlation/precision across the five labs for each interval. (B) Visualizing the number of probe sets in each interval between the different probe set definitions.

The decrease in precision for probe set definitions to ensEMBL exons was likely due to the fewer number of probes that map to each exon (compared to the whole transcript). We therefore calculated the mean number of probes mapping to each probe set using the different probe set definitions. Indeed, the mean number of probes per probe set is lower for ensEMBL exons (Table 2). Using fewer probes when estimating an expression level likely increase the variance and lower the precision. Likewise, the improved precision for the other updated probe set definitions could be due to a larger number of probes mapping to each probe set since the mean number of probes are higher than for the original probe set definitions (Table 2). We therefore analyzed the precision as a function of the number of probes used to estimate each probe set (Figure 1a) for all probe set definitions and averaged across the five labs. To enable this analysis we had to group probe sets in bins of 4 as too few probe sets would otherwise give unreliable precision estimates. We found a positive correlation between number of probe sets and precision. However, the updated probe set definitions appears to achieve better precision than the original, even when similar numbers of probes were integrated into the signal estimates (see probe intervals 10–13 and 14–17 in Figure 1a). The numbers of probe sets defined by a particular number of probes are presented in Figure 1b. Similar results were obtained when analyzing the data from each of the five labs independently [see Additional file 1]. Thus, updated probe set definitions have significant improvements in precision.

**Table 2:** Characteristics of probe set definitions

**Probe set definition**	**Number of probe sets**	**Mean number of probe pairs per probe set**
NetAffx (original)	22283	11.1
ensEMBL exon	35191	9.3
ensEMBL gene	18671	14.0
ensEMBL transcript	36174	13.9
Entrez	12132	14.1
RefSeq	17880	14.9
UniGene	11694	15.0

The table shows the number of probe sets together whith the average number of probes within each probeset obtained using each probe set definition for the HG-U133A platform.

### Significant improvement in accuracy using updated probe set definitions

**Figure 2:**
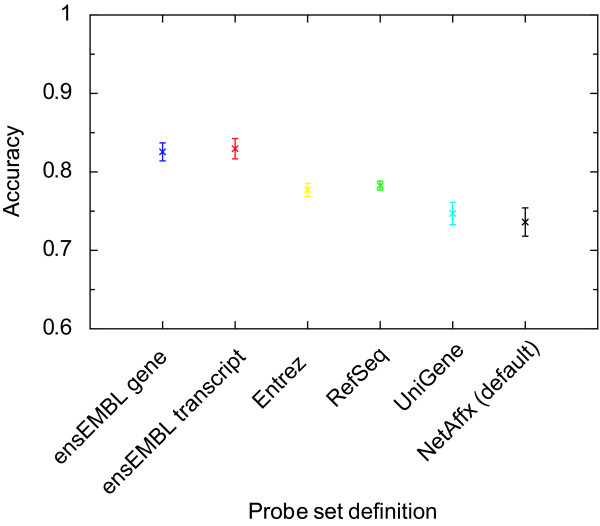
**Improved accuracy using updated probe set definitions**. The accuracy between measured log2 ratios using RT-PCR and microarrays were compared for microarray measurements using the original (NetAffx) and updated probe set definitions. The accuracy was obtained by calculating the slope from a linear regression comparing the obtained log2 ratios from the microarray to those estimates obtained using RT-PCR, where the microarray estimates were obtained using the different probe set definitions. A slope of 1 would indicate perfect accuracy. The mean accuracy across five different labs is plotted together with the standard deviations.

We next investigated the accuracy in detecting differentially expressed genes when using the updated probe set definitions. Accuracy was defined (ref11) to estimate how close the microarray estimates are to the "real expression" changes. Most often the "real" expression is measured using RT-PCR (real time PCR). To assess how accurate estimates the updated probe set definitions achieved, we compared the differential expression detected with microarrays to those measured by RT-PCR for 16 genes [11], for the different probe set definitions respectively. The accuracy was defined as the slope after a linear regression [11] between RT-PCR and microarray data (i.e. an accuracy of 1.0 is optimal,). We calculated the difference in accuracy for each lab between the updated probe set definitions and the standard probe set definition and then asked if the mean accuracy difference (averaged across the five labs) was significant using a paired t-test (two-tailed distribution). Significant improvements in accuracy were observed (when data was normalized using RMA) when all but the UniGene definition. The mean accuracy differences between the updated probe set definitions and the standard probe set definition, as well as the p-values calculated using the paired t-test are shown in Table 3. The slopes estimated from the five different labs were in general in good agreement as evident by the low standard deviations in Figure 2 and Table 3.

**Table 3:** Improved accuracy using updated probe set definitions

**RMA**	**Mean Slope**	**p-value**	**Std**
**NetAffx (original)**	0.74		0.02
ensEMBL gene	0.83	0.00040	0.01
ensEMBL transcript	0.83	0.00053	0.01
Entrez	0.78	0.00430	0.01
RefSeq	0.78	0.00381	0.01
UniGene	0.75	0.07085	0.01

The accuracy between measured log2 ratios using RT-PCR and microarrays were compared for microarray measurements using the original (NetAffx) and updated probe set definitions across 16 genes. The accuracy was obtained by calculating the slope from a linear regression comparing the obtained log2 ratios estimates between the sample groups from the microarray to those estimates obtained using RT-PCR, where the microarray estimates were obtained using the different probe set definitions. A slope of 1 would indicate perfect accuracy.

The table shows the mean of the obtained accuracy across the five labs. We performed a t-test to assess the difference in means of accuracy between the updated probe set definitions and the original probe set definition (NetAffx); p-values were calculated using a paired t-test (two-tailed distribution). The standard deviation for the mean is also shown. The data represents an assessment of RMA normalized data.

## Discussion

Accurate probe set definitions are essential for integrating the probe signals from a microarray experiment into a set of expression levels. Different investigators have recently introduced updated probe set definitions [7,8] that more accurately map probes to genes and transcripts. The updated probe set definitions for Affymetrix arrays use fewer or more probes (by removing erroneous or non-specific probes and by pooling several probe sets targeting the same gene/transcript) but also estimates fewer probe sets (i.e. transcripts or genes) as compared with the original annotations. As a consequence, the number of probe pairs per probe set is no longer identical across all probe sets. We therefore investigated how the updated probe set definitions with variable number of probe pairs integrated into each probe set estimate would affect the precision and accuracy in estimated expression levels. We initially hypothesized that that the more stringent selection of probes that would be included in each probe set may have a negative impact on precision as fewer probes would be included in some probe sets. Such a result would have argued for caution in using updated probe set definitions.

We show that using updated probe set definitions (custom CDFs) improves both the precision and accuracy of the relative expression level estimates. The improvement in precision depends mainly on the increased number of probe pairs per probe set (Figure 1a). Furthermore, an improvement was also detected in comparisons where similar number of probe pairs were used which indicate that the re-annotation improves the expression estimate presumable by removing erroneous or non-specific probes that otherwise adds noise. The observed improvement in accuracy may also be due to removal of erroneous probes that otherwise would lower the estimated differential expression estimate. Improving the precision and accuracy effect the possible inferences from an experiment. E.g. a microarray study with increased precision will likely improve the ability to identify differential expressed genes, due to a lower variation within the biological groups. Similarly, the improvement in accuracy leads to prediction of relative expression level changes that better reflect the 'real change' (as measured by more precise methods). This is the first assessment on the impact of updated probe set definitions (and custom CDFs) on these two fundamental measurements and our results strongly argue for a wide spread use of updated probe set definitions.

More accurate probe set definitions will also be important for studies comparing microarray expression levels to sequence features, e.g. on pre-mRNAs. The correct mapping of pre-mRNA sequences to expression levels will likely improve the possible inferences (e.g. the identification of cis-regulatory elements). Therefore, we predict that using updated probe set definitions will be important for studies on post-transcriptional regulation [12] e.g. at the level of miRNA targets (e.g. [13]) and alternative splicing.

The public repositories (e.g. Gene Expression Omnibus [14], ArrayExpress [15] and Stanford Microarray Database [16]) contain a wealth of gene expression data that could be used for re-analysis and meta analysis [4]. Only experiments that are deposited as raw data however could be re-analyzed by taking advantage of the updated probe set definitions. It is therefore troublesome that still only a limited portion of the data in the public repositories are available as raw data [17] to be used for future comparative microarray analysis e.g. using updated probe set definitions.

## Conclusion

Updated probe set definitions do not only offer expression levels that are more accurately associated to genes and transcripts but also shows improvements in the estimated transcript expression levels. These results give further support for a widespread use of updated probe set definitions for analysis and meta-analysis of microarray data.

## Methods

### Gene expression data

We re-analyzed the gene expression data generated in five different labs using the same RNA hybridized to HG-U133A Affymetrix arrays [11]. The raw data files (i.e. CEL files) were downloaded from the authors' URL [18]. Each lab produced a comparison two different samples in duplicates.

We downloaded the version 7 of the updated probe set definitions [7] generated for the HG-U133A platform from the authors' URL [19]. We considered all seven probe set definition that mapped probes to ensEMBL exons (Hs133A_Hs_ENSE_7), ensEMBL transcripts (Hs133A_Hs_ENST_7), ensEMBL genes (Hs133A_Hs_ENSG_7), Entrez Gene IDs (Hs133A_Hs_ENTREZG_7), RefSeq (Hs133A_Hs_REFSEQ_7) and UniGene (Hs133A_Hs_UG_7).

Probe set summaries were calculated for each laboratory (4 arrays per lab) using three different methods for expressional level estimation (MAS5, RMA and GCRMA) and seven different custom CDF files, resulting in twenty-one different probe set estimates per array. All calculations were performed in R using the bioconductor packages affy and gcrma and the default settings for MAS5, RMA and GCRMA. We named the custom CDF files as previously described [7].

### Precision

The data sets generated in each lab consisted of sample A hybridized to two arrays, A1 and A2 and sample B hybridized to two arrays B1 and B2. Following Irizarry and co-workers [11], precision was defined as the Pearson correlation between the log2 ratios of A1/B1 and A2/B2. The precision presented in Table 1 was calculated on MAS5, RMA and GCRMA estimated probes sets signals and using the different custom CDF files independently. Figure 1 shows the precision as a function of the number of probes integrated into a probe set for the GCRMA generated expression levels.

### Accuracy

The relative change in expression levels of 16 genes were previously measured by RT-PCR [11] and we downloaded the corresponding log2 ratios from the authors' webpage [18]. The accuracy measures how the magnitude of differential expression on a specific platform compares to the difference obtained by a more precise method e.g. RT-PCR. We used the annotations from NetAffx [20] to map the probe set of these 16 genes to the different updated probe set definition identifiers [see Additional file 2]. The accuracy was defined as the slope between the RT-PCR and microarray log2 ratios, determined by a linear regression [11]. The accuracy for the different custom CDF files on RMA expression values are presented in Table 2. Accuracy estimates using GCRMA [see Additional file 3] were also calculated.

## Authors' contributions

RS and OL designed and performed the experiment and wrote the manuscript. Both authors read and approved the final manuscript.

## Supplementary Material

Additional file 1 **Precision for the data from each laboratory**. Similarly to main figure 1, we compared the precision in microarray experiments when using the updated probe set definitions in comparison with the original one for each lab independently. We measured the precision as the Pearson correlation between the log2 ratios of expression levels obtained using GCRMA. The x-axis show the number of probes integrated into each probe set. We grouped the number of probes into intervals of four in order to obtain a sufficiently large number of probe sets to get a robust estimate of each data point. The grouping is identical to Figure 1a. Click here for file

Additional file 2 **RT-PCR Map**. A table (cvs file) with the identifiers used to associate the RT-PCR data from ref. 11 to the expression estimates derived using different updated probe set definitions. Click here for file

Additional file 3 **Accuracy for GCRMA data**. Table with accuracy estimates for the different updated probe set definitions using GCRMA data. All tests were performed as described for Table 3. Click here for file
